# Phonemic restoration of interrupted locally time-reversed speech: Effects of segment duration and noise levels

**DOI:** 10.3758/s13414-021-02292-3

**Published:** 2021-04-13

**Authors:** Kazuo Ueda, Valter Ciocca

**Affiliations:** 1 Department of Human Science/Research Center for Applied Perceptual Science/Research and Development Center for Five-Sense Devices, Kyushu University, 4-9-1 Shiobaru, Minami-ku, 815-8540 Fukuoka Japan; 2 School of Audiology and Speech Sciences, The University of British Columbia, 2177 Wesbrook Mall, Vancouver, BC V6T 1Z3 Canada

**Keywords:** Interrupted speech, Locally time-reversed speech, Noise filling, Phonemic restoration, Speech intelligibility

## Abstract

Intelligibility of temporally degraded speech was investigated with locally time-reversed speech (LTR) and its interrupted version (ILTR). Control stimuli comprising interrupted speech (I) were also included. Speech stimuli consisted of 200 Japanese meaningful sentences. In interrupted stimuli, speech segments were alternated with either silent gaps or pink noise bursts. The noise bursts had a level of − 10, 0 or + 10 dB relative to the speech level. Segment duration varied from 20 to 160 ms for ILTR sentences, but was fixed at 160 ms for I sentences. At segment durations between 40 and 80 ms, severe reductions in intelligibility were observed for ILTR sentences, compared with LTR sentences. A substantial improvement in intelligibility (30–33%) was observed when 40-ms silent gaps in ILTR were replaced with 0- and + 10-dB noise. Noise with a level of − 10 dB had no effect on the intelligibility. These findings show that the combined effects of interruptions and temporal reversal of speech segments on intelligibility are greater than the sum of each individual effect. The results also support the idea that illusory continuity induced by high-level noise bursts improves the intelligibility of ILTR and I sentences.

**Supplementary Information:**

The online version contains supplementary material available at 10.3758/s13414-021-02292-3.

## Introduction

In describing the temporal characteristic of speech signals, Rosen (1992) proposed a framework according to which the temporal structure of speech is organized into three features: envelope (2–50 Hz), periodicity (50–500 Hz) and fine structure (600–10,000 Hz). The idea that different features of the speech signal are concurrently processed within time windows of different duration was incorporated into the “two-temporal-windows integration” (2TWI) model by Poeppel and his colleagues (Poeppel, 2003; Sanders and Poeppel, 2007; Giraud & Poeppel, 2012; Chait, Greenberg, Arai, Simon, & Poeppel, 2015; Teng, Tian, & Poeppel, 2016; Teng & Poeppel, 2020). According to this model, two temporal windows are applied concurrently by the auditory system for integrating auditory information over time: a short temporal window (${\sim }20$–30 ms), and a long temporal window (${\sim }200$ ms). The short temporal window is used to extract information that is represented within a rapid time scale—such as segmental, phoneme-level information in speech. The long temporal window is used to represent information that varies over a longer time scale, such as syllable-size units in speech. The model has received support from both neurological evidence about cortical activity (Giraud & Poeppel, 2012; Luo & Poeppel, 2007; Teng & Poeppel, 2020) as well as behavioral studies employing both speech (Chait et al., 2015) and nonspeech stimuli (Sanders & Poeppel, 2007; Teng et al., 2016).

**Fig. 1:**
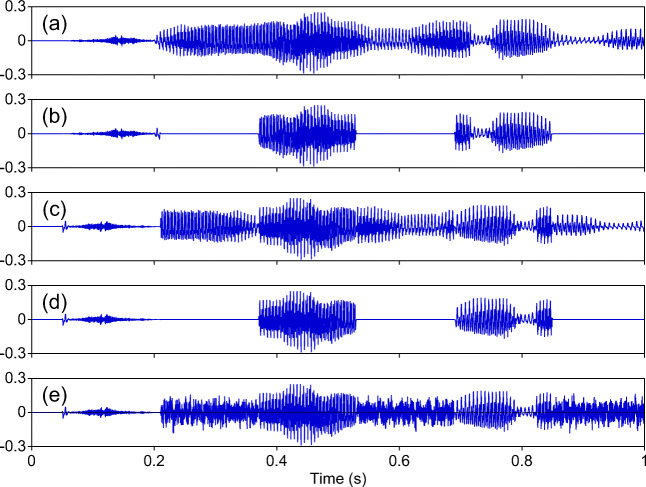
Examples of stimulus waveforms. **a** Original speech waveform. **b** Interrupted speech (I) of 160-ms on and off time. **c** Locally time-reversed speech (LTR) of 160-ms segment duration. **d** Interrupted locally time-reversed speech (ILTR) of 160-ms segment duration. **e** Interrupted locally time-reversed speech of 160-ms segment duration with pink noise of 0 dB (ILTR_N0)

A useful paradigm for testing the predictions of the 2TWI model for speech processing is the so-called “locally time-reversed” (LTR) speech (Steffen & Werani, 1994; Saberi & Perrott, 1999; Greenberg & Arai, 2004; Kiss, Cristescu, Fink, & Wittmann, 2008; Stilp, Kiefte, Alexander, & Kluender, 2010; Remez et al., 2013; Ishida, Samuel, & Arai, 2016; Ueda, Nakajima, Ellermeier, & Kattner, 2017; Ishida, Arai, & Kashino, 2018; Nakajima, Matsuda, Ueda, & Remijn, 2018; Teng, Cogan, & Poeppel, 2019). To produce LTR speech, sentences are first segmented at regular intervals, and then each segment is temporally reversed; finally, the time reversed segments are concatenated (see Fig. 1c). Segment duration is one of the key predictors of the intelligibility of LTR speech. Stilp, Kiefte, Alexander, and Kluender (2010) found that, for speech produced at a moderate speech rate, LTR was perfectly intelligible when segment duration was shorter than about 40 ms. Ueda, Nakajima, Ellermeier, and Kattner (2017) replicated this finding with LTR sentences in four languages (English, German, Japanese, Mandarin) when speech rates were normalized. They reported that intelligibility was at ceiling for all languages with 20-ms segments, remained high (about 90% correct or higher) when segment duration was shorter than 45 ms, and then declined to about 40%-50% as segment duration was increased further to 70 ms. According to the 2TWI model, the high intelligibility of LTR speech comprised of segments shorter than about 40 ms results from the preservation of the segmental content of the sentences through temporal integration within short temporal windows. When segmental information is preserved, accurate envelope information can also be recovered through temporal integration over long time windows. By contrast, with segment duration is longer than 40 ms and up to 70 ms, segmental information is degraded but the intelligibility of LTR speech remained relatively high likely because envelope features such as manner, segmental/syllable duration, and speech rhythm can still be retrieved by temporal integration over long time windows.

While the results of studies on LTR speech are consistent with the predictions of the 2TWI model, it is unclear to what extent accurate segmental information is required to extract envelope information. To investigate this question, the present study employed LTR sentences in which segments were periodically replaced by silent intervals (ILTR stimuli; see Fig. 1d). When half of the speech signal is physically removed, and the remaining speech is intact, intelligibility is high (80% correct or higher) for segment durations up to about 100 ms (Miller & Licklider, 1950; Licklider & Miller, 1951; Powers & Speaks, 1973). We reasoned that if the extraction of envelope information from long time windows requires that segmental information is partially preserved then, as segment duration increases beyond 20-30 ms, intelligibility should be reduced in ILTR speech relative to non-time-reversed sentences. Intelligibility for LTR speech should be higher than for ILTR sentences, but lower than with non-time-reversed sentences because the segmental information in the remaining speech signal of the latter sentences is intact. If envelope information can be retrieved in spite of compromised segmental information, intelligibility should be equally high for ILTR, LTR, and non-time-reversed interrupted sentences when segment duration is longer than $\sim $20-30 ms. Finally, with 20-ms (or shorter) segments, intelligibility for ILTR and LTR, and non-time-reversed sentences should be equally high because at this segment duration both segmental and envelope cues would be well preserved for all sentence types.

A second question investigated in the current study concerns the role of the linguistic context on intelligibility of degraded speech using the phonemic restoration paradigm. The processing of speech units such as syllables and words within interrupted sentences has been shown to be affected by both the acoustical and linguistic context. Several studies showed that replacing silent gaps with noise could improve the intelligibility of meaningful sentences (Cherry & Wiley, 1967; Holloway, 1970; Powers & Wilcox, 1977; Bashford, Riener, & Warren, 1992; Wiley, 1968). For example, filling silent gaps (1.5-Hz interruption cycle, i.e., 333-ms gaps) with noise bursts that had the same level as the speech signal produced a 15% increase in intelligibility (Powers & Wilcox, 1977). However, intelligibility did not improve when speech segments within lists of isolated words were replaced by noise bursts (Miller & Licklider, 1950). The perceptual restoration of missing speech signals that are replaced by noise whose level is equal or higher than the adjacent signal is known as “phonemic restoration” (Warren, 1970; Bashford & Warren, 1987; Warren, Bashford, Healy, & Brubaker, 1994). Warren (2008) proposed that the perceptual (phonemic) restoration of interrupted speech involves two stages: First, illusory continuity (temporal induction) processes generate the percept of a continuous (uninterrupted) signal. These processes apply to verbal and nonverbal sounds alike, and require specific stimulus conditions to generate continuous percepts. One important requirement is that the interposed noise bursts should be intense enough to mask the signal completely if the signal were actually present (Warren, Obusek, & Ackroff, 1972; Houtgast, 1972). In addition, spectral similarity of the interposed noise to an original speech (Samuel, 1981a, b) has been shown to affect the magnitude of phonemic restoration. Second, speech recognition takes place and, if sufficient linguistic context is available, sentence intelligibility is enhanced when the speech signal is interrupted by noise bursts rather than by silent gaps.

In the present investigation, local time-reversal was combined with periodic interruptions consisting of silent or noise intervals to determine whether contextual information can compensate for the degradation of segmental and envelope information. When the silent gaps in ILTR speech are replaced by noise (ILTR_N), intelligibility may be enhanced in comparison with ILTR speech of the same segment durations, given the effect of linguistic context reported in previous studies of phonemic restoration. To test this prediction, we compared the intelligibility of meaningful ILTR and ILTR_N sentences by varying segment duration and the level of the noise in ILTR_N sentences.

## Method

### Participants

Twenty Japanese native speakers (ten females and ten males; mean age = 21.4, SD = 1.31) with normal hearing (screened at a level of 25 dB HL at octave frequencies from 250 to 8000 Hz, using an audiometer, Rion, AA-56, Rion Co., Ltd., Kokubunji, Japan) participated. Ten listeners were allotted to conditions with female speech stimuli, whereas the other ten to male speech. The research was conducted with prior approval of the Ethics Committee of Kyushu University (approval ID: 70).

### Stimuli, conditions, and procedure

Two hundred Japanese sentences spoken by both a female and a male speaker were extracted from the “Multilingual Speech Database 2002” (NTT Advanced Technology Corp., Kawasaki, Japan). There were five types of stimuli (interrupted, interrupted-with-noise, LTR, ILTR, and ILTR_N). In interrupted (I) stimuli, every other segment was replaced with a silent period of the same duration (Fig. 1b). In interrupted-with-noise (I_N) stimuli, the silent segments were replaced by pink noise bursts. By replacing silent gaps with pink noise samples, this study investigated the role of linguistic context by comparing interrupted sentences with and without noise bursts so that in both conditions the available speech signal was not masked by a simultaneous noise burst. Pink noise rather than white noise (or other intervening signal) was used because pink noise has a closer spectral slope to a speech spectrum, and because spectral similarity has been shown to affect the magnitude of phonemic restoration (see Samuel 1981a, b). In LTR stimuli, each segment was reversed in time (Fig. 1c). In ILTR stimuli, every other LTR segment was replaced with a silent period of the same segment duration (Fig. 1d). In ILTR_N stimuli, the silent segments were replaced by pink noise bursts (Fig. 1e). For both I and I_N stimuli, segment duration was fixed at 160 ms, because our preliminary results (Ueda et al., 2017) showed that the differences in intelligibility between I and I_N + 6 stimuli changed from 0% to 10% over the 30–210-ms range of segment duration. Segment duration was either 20, 40, 60, 80, or 160 ms for the LTR, ILTR, and ILTR_N stimuli. Other details of the stimuli and conditions are described in the Supplemental Material.

The stimuli were presented to participants using custom software written with the LiveCode package (LiveCode, 2018). One hundred and forty-five sentences were randomly selected for each participant from the 200 sentences produced by one of the speakers. These sentences were randomly allotted to one of 29 conditions (five sentences for each condition). Since the average number of morae (syllable-like units in Japanese) per sentence was 18, each condition contained 90 morae on average for each participant. The stimuli were presented to participants diotically through headphones (Beyer, DT 990 PRO, Beyerdynamic GmbH, Heilbronn, Germany) in a sound-attenuated booth (Music cabin, SD3, Takahashi Kensetsu, Kawasaki, Japan). The level of speech was adjusted to 60 dB SPL, using a 1-kHz calibration tone provided with the speech database. The sound pressure levels were measured with an artificial ear [Brüel & Kjær, type 4153 (Brüel & Kjær Sound & Vibration Measurement A/S, Nærum, Denmark)], a condenser microphone (Brüel & Kjær, type 4192), and a sound level meter (Brüel & Kjær, type 2260). Each stimulus was presented three times with 0.5-s inter-stimulus-intervals within a trial, following the procedure previously used by Ueda et al., (2017). Participants were instructed to write down exactly what they heard with hiragana or katakana (sets of symbols that are used to represent Japanese morae) without guessing. The listeners were instructed to write down the morae they could clearly recognize, and to omit any morae that they would have had to guess.

## Results

**Fig. 2:**
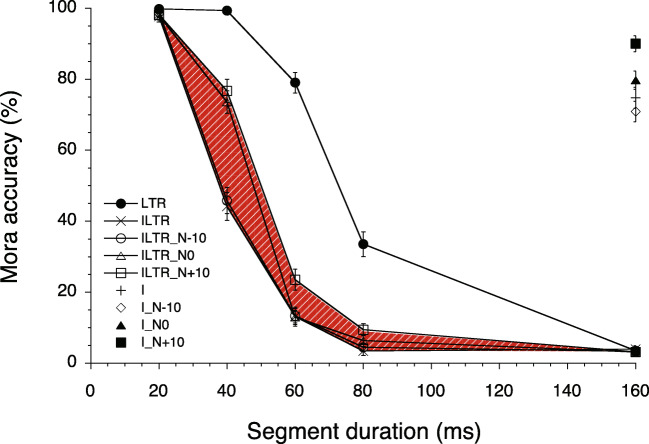
Mean percentage of mora accuracy as a function of segment duration, types of degradation, and noise levels for the following conditions: LTR (locally time-reversed speech); ILTR (interrupted locally time-reversed speech); ILTR_N − 10 (interrupted LTR with noise of − 10 dB); ILTR_N0 (with noise of 0 dB); ILTR_N + 10 (with noise of + 10 dB); I (interrupted speech); I_N − 10 (interrupted speech with noise of − 10 dB); I_N0 (with noise of 0 dB); I_N + 10 (with noise of + 10 dB). The interruption introduced in the LTR stimuli caused a substantial decrease in intelligibility (see the difference between the LTR and ILTR curves). The *shading in red* (online) shows the area between the ILTR_N + 10 and ILTR curves to illustrate the effect of the presence of noise. The *error bars* represent SEM

The data of one participant were not included in the data analysis due to a failure to perform the task as instructed: the participant used Chinese characters in combination with hiragana symbols, instead of using hiragana and katakana symbols. The percentage of correct morae for the remaining listeners (*N* = 19) are shown in Fig. 2.

Performance was at ceiling for all conditions with 20-ms segments, and at floor level with 160-ms segments for the LTR, ILTR, ILTR_N − 10, ILTR_N0, and ILTR_N + 10 conditions. The mean accuracy for the ILTR and ILTR_N − 10 conditions decreased sharply from the 20-ms segment condition (98% and 99%, respectively) to the 40-ms segment duration (44% and 46%), and dropped close to the performance floor with 60-ms (both at 13%) and 80-ms segments (3% and 4%, respectively). When segment duration was 40 ms, performance for both the ILTR_N0 and ILTR_N + 10 conditions was 74% and 77%, respectively. At the 60-ms segment duration, only the highest noise level (+ 10 dB) resulted in a relatively small (11%) improvement in intelligibility relative to the ILTR condition. Finally, intelligibility for the LTR condition was still at ceiling with 40-ms segments, and was consistently higher than all of the ILTR conditions with 60- and 80-ms segments.

A logistic regression model was selected because the data were dichotomous in nature (counts of correct vs incorrect responses for each mora), and therefore are more accurately modelled using a binomial likelihood instead of the normal likelihood that is assumed by linear models such as an analysis of variance (ANOVA). The disadvantages of using an ANOVA on percent correct scores (whether untransformed or arcsine-transformed) include lesser interpretability and lower power (see, for example, Warton and Hui (2011)). A beta-binomial likelihood was selected in order to minimize the risk of type I errors that can occur with overdispersed data. As an estimate of effect size, the area under the curve (AUC) is reported.

Multiple beta-binomial regression analyses were performed for the data in Fig. 2, except for those in the I and I_N conditions, which were analyzed separately. All statistical effects had a *p* level smaller than .001, unless reported otherwise. One multiple regression model was used to estimate the effect of introducing silent interruptions in ILTR speech. This model comprised duration (20, 40, 60, 80, and 160 ms), type (LTR and ILTR), and the duration by type interaction as predictors. The full model resulted in a large effect size [AUC = .94; main effect of segment duration, Wald *χ*^2^(4) = 321.19; main effect of stimulus type, Wald *χ*^2^(1) = 4.35, *p* = .037; duration by type interaction effect, Wald $\chi ^{2}{\kern -.3pt}({\kern -.3pt}4{\kern -.3pt}) = 1{\kern -.3pt}0{\kern -.3pt}7{\kern -.3pt}.{\kern -.3pt}8{\kern -.3pt}3$]. To estimate differences between ILTR and ILTR_N conditions, a multiple beta-binomial regression model that included duration (20, 40, 60, 80, and 160 ms), noise level (silent, − 10, 0, and + 10 dB), and the duration by noise level interaction as predictors was applied to the data. The full model resulted in a large effect size [AUC = .92; main effect of duration, Wald *χ*^2^(4) = 494.73; main effect of noise level, Wald *χ*^2^(3) = 1.42, *p* = .70; duration by noise level interaction, Wald *χ*^2^(12) = 59.68]. The duration by noise level interaction shows that substituting silent gaps with noise improved intelligibility for ILTR sentences, though not for all segment durations and noise levels. Specifically, in the 40-ms segment condition, the presence of 0- and + 10-dB noise improved accuracy by 30% and 33%, respectively [simple effect of noise level, Wald *χ*^2^(3) = 59.91; small effect size, AUC = .66]. An 11% improvement in accuracy was observed in the + 10-dB condition for the 60-ms segments [simple effect of noise level, Wald *χ*^2^(3) = 14.73, *p* < .002; small effect size, AUC = .58]. Slight increments in accuracy were observed with 0-dB noise (3%) and + 10-dB noise (6%) for the 80-ms segments [simple effect of noise level, Wald *χ*^2^(3) = 17.13; small effect size, AUC = .60].

Intelligibility was moderate with forward playback of sentences interrupted every 160 ms (75%; I condition). The addition of low- (− 10 dB) or equal-level (0 dB) noise did not improve the intelligibility of interrupted sentences. By contrast, an intelligibility improvement of 15% was observed when noise of the highest level (+ 10 dB) replaced the silent gaps in the interrupted sentences.

A beta-binomial regression analysis was performed for the I and I_N conditions. There was a small main effect of noise level, Wald *χ*^2^(3) = 30.4, *p* < .001, AUC = .61. A multiple comparison with Dunnett’s test between the + 10-dB condition and the silent gap (I) condition resulted in *p* < .001; the *p* values for other comparisons with the I condition exceeded .40.

## Discussion

In summary, a reduction in intelligibility was observed for ILTR sentences, compared to LTR sentences, with segment durations of 40 to 80 ms. A substantial improvement in intelligibility was observed with ILTR sentences when pink noise was alternated with 40-ms segments (0 and + 10 dB conditions). The replacement of interruptions with the + 10 dB noise bursts with 60-ms segments resulted in a small improvement relative to the equivalent ILTR condition. For segment durations of 40 ms or longer, performance in the ILTR_N-10 and ILTR conditions was equally poor. At the 20-ms segment duration, performance was at ceiling for all sentence types. The combined effects of interruption and LTR on intelligibility were greater than the sum of each individual effect.

The finding that intelligibility was at ceiling for 20-ms segments is in agreement with the results obtained by Teng, Tian, and Poeppel (2016). They investigated temporal integration using sequences of rising or falling frequency glides. When their listeners were asked to discriminate pairs of such sequences on the basis of the differences in the direction of the frequency glides (“local” task) performance was generally poor when glides lasted 15–20 ms. This result suggests that listeners treated rising and falling glides as perceptually equivalent when processed within a short temporal window (about 20 ms). Together with the results of the present study, this evidence leads us to speculate that the auditory system might integrate spectral information in a way that is analogous to performing short-term FFT spectra over a short temporal window of about 20 ms. Further research is warranted to determine whether such spectral averaging within short temporal windows is a generally applicable finding.

The present results showed that introducing periodic interruptions in LTR speech lowered intelligibility when segment duration was between 40 and 80 ms. Specifically, the difference in accuracy between the LTR and ILTR conditions was about 55% at the 40-ms segment duration. Since the intelligibility of non-time-reversed interrupted speech with a 0.5 STF is almost perfect at this segment duration (Powers & Speaks, 1973), the present evidence is consistent with the idea that preserved segmental information obtained from short time windows is required to recover envelope features that are extracted from long time windows. If segmental information is processed within short ($\sim $20–30 ms) temporal windows, then one would predict that segmental information is largely preserved for 40-ms LTR sentences, and for 20-ms ILTR sentences. The current finding that intelligibility was relatively high for these conditions supports this prediction. The extraction of envelope features should also be accurate for these conditions according to the conjecture that long temporal windows integrate information over several, successive short temporal windows. Therefore, the accurate representation of segmental features would result in a precise representation of the dynamic amplitude changes that characterize envelope cues. By contrast, the representation of segmental features would be degraded when LTR segments are lengthened to 60 ms or longer because the temporal order of the spectral information captured within short temporal windows would be reversed. A similar disruption in the representation of segmental information would occur with ILTR sentences with 40-ms or longer segments. Envelope information would also be represented inaccurately if it is derived from degraded segmental information extracted from successive short temporal windows. This explanation is consistent with the drastically reduced intelligibility of LTR speech with 40 ms or longer segments, and with ILTR sentences whose segments are longer than 20 ms. Within the framework of the 2TWI model proposed by Poeppel and colleagues (Chait et al., 2015; Giraud & Poeppel, 2012; Teng et al., 2016; Teng & Poeppel, 2020), the current findings suggest that the processing of global (envelope) cues is dependent on the retrieval of segmental information within short time windows (local cues). In other words, the processing of envelope information over long temporal windows may not be independent of the processing of segmental information within short temporal windows.

Replacing silent gaps with pink noise whose level was 0 or + 10 dB relative to the speech level improved intelligibility in ILTR sentences whose segments had a duration of 40 ms. Since performance was equally poor for the ILTR and the ILTR_N − 10 conditions, the improvement in the recognition of ILTR_N0 and ILTR_N + 10 sentences is likely to be associated with the phenomenon of phonemic restoration. Following Warren (2008)’s two-stage model, we hypothesize that at this segment duration the presence of illusory auditory continuity (stage 1) facilitated the recovery of segmental and/or envelope information, thereby providing sufficient contextual information for the occurrence of phonemic restoration (stage 2). Whether auditory continuity selectively affects the recovery of segmental or envelope information is a question that requires further investigation. Large effects of the presence of 0 and + 10 dB noise bursts were observed with 40-ms segments but not with longer segments in ILTR sentences. By contrast, the presence of high-level noise bursts has been shown to enhance the recognition of natural sentences for interruptions as long as 333 ms (Powers and Wilcox, 1977). Although it is plausible that illusory auditory continuity occurred in all the ILTR_N0 and ILTR_N + 10 conditions of the present study, it is likely that neither local nor global cues could be used effectively for recovering contextual information when segment duration was longer than about 60 ms. As a consequence, no phonemic restoration and poor intelligibility were observed for these stimuli.

In conclusion, the current findings provide preliminary evidence that the extraction of envelope information from long temporal windows is conditional on the integrity of segmental information extracted from short temporal windows. Secondly, we found that phonemic restoration only occurred with locally time-reversed sentences whose segments were shorter than 60 ms. While the present findings are consistent with the framework of the 2TWI model, they also indicate the need for future investigations about the interdependence of the processing of speech information in different temporal windows. Future studies may also explore the extent to which segmental and envelope cues support the use of linguistic context for speech recognition with temporally degraded speech signals.

## Electronic supplementary material

Below is the link to the electronic supplementary material.

(PDF 24.6 KB)
