# Supplementary material for: Phonemic restoration of interrupted locally time-reversed speech: Effects of segment duration and noise levels
Source: Atten Percept Psychophys. 2021 Apr 13;83(5):1928–34. doi: 10.3758/s13414-021-02292-3 (PMC8213671; doi:10.3758/s13414-021-02292-3)
Supplement: Supplementary file 1 — (PDF 24.6 KB) [file 13414_2021_2292_MOESM1_ESM.pdf]

Attention, Perception, and Psychophysics manuscript No.
(will be inserted by the editor)

Supplemental Material: Phonemic restoration of interrupted locally time-reversed speech

Effects of segment duration and noise levels

Kazuo Ueda · Valter Ciocca

Received: date / Accepted: date

1 Details of Stimuli and Conditions

Two hundred Japanese sentences spoken by both a female and a male speaker were extracted from the "Multilingual Speech Database 2002" (NTT Advanced Technology Corp., Kawasaki, Japan). The average number of morae per sentence was 18, with a standard deviation of 2.9. The sentences in the database were based on the sentences chosen from articles in newspapers and magazines. These sentences were originally recorded with a 16-kHz sampling rate and 16-bit quantization, and were converted to a 44.1-kHz sampling rate and 16-bit quantization using Praat (Boersma & Weenink, 2016). Sentences were subdivided into equal-duration segments. The last segment of each segmented sentence typically contained a silent portion whose duration varied depending on the overall duration of the sentence and on segment duration. There were five types of stimuli (Interrupted, Interrupted-with-Noise, LTR, ILTR, and ILTR_N). In Interrupted (I) stimuli, every other segment was replaced with a silent period of the same duration (Fig. 1b). In Interrupted-with-Noise (I_N) stimuli, the silent segments were replaced by pink noise bursts. In LTR stimuli, each segment was reversed in time (Fig. 1c). In ILTR stimuli, every other LTR segment was replaced with a silent period of the same segment duration (Fig. 1d). In ILTR_N stimuli, the silent segments were replaced by pink noise bursts (Fig. 1e).

For both I and I_N stimuli, segment duration was fixed at 160 ms, because our preliminary results (Ueda et al., 2017) showed that the differences in intelligibility between I and I_N+6 stimuli changed from 0 to 10% over the 30–210-ms range of segment duration. Segment duration was either 20, 40, 60, 80, or 160 ms for the LTR, ILTR, and ILTR_N stimuli. The duration of 60 ms was included because our preliminary results (Ueda et al., 2017) showed rapid transition of the performance around this duration. For the I_N and the ILTR_N stimuli, a band-passed pink noise sample (80 and 8000 Hz cut-off frequencies) was synthesized for each sentence; the pink noise sample had the same length as the segmented speech file. The root-mean-square amplitude of the noise was adjusted to either -10, 0, or $+10$ dB relative to the average root-mean-square amplitude of each of the original spoken sentences. Then each pink noise sample was segmented periodically and mixed with the respective, segmented speech sentences such that speech and noise segments alternated with each other in I_N and ILTR_N stimuli. No noise segment was used twice across all stimuli. For both speech and noise samples, segment duration included 2.5-ms rise and fall cosine ramps. To summarize, there was one segment duration (160 ms) for the I stimuli, one

K. Ueda
Department of Human Science/Research Center for Applied Perceptual Science/Research and Development Center for Five-Sense Devices, Kyushu University, 4-9-1 Shiobaru, Minami-ku, 815-8540 Fukuoka, Japan
Tel.: +81-92-553-9460
Fax: +81-92-553-9460
E-mail: ueda@design.kyushu-u.ac.jp

V. Ciocca
School of Audiology and Speech Sciences, The University of British Columbia, 2177 Wesbrook Mall, Vancouver, BC V6T 1Z3, Canada

segment duration and three noise levels for the I_N stimuli, five segment durations for each of the LTR and ILTR stimuli, and five segment durations by three noise levels for the ILTR_N stimuli, for a total of 29 conditions. The stimuli were generated using a custom software written in the J language (J Software, 2016).

References

Boersma, P., & Weenink, D. (2016). *Praat: Doing phonetics by computer [computer program].* (Version 6.0.21, http://www.praat.org/, (Last viewed 9 November 2016))

J Software. (2016). *The J programming language [computer language].* (Version J64 806, http://www.jsoftware.com/, (Last viewed 12 June 2019))

Ueda, K., Inui, N., Shiraki, K., Ciocca, V., Nakajima, Y., & Gemijn, G. B. (2017). Perceptual restoration of interrupted locally time-reversed speech. In K. Ueda, H. Ito, G. B. Remijn, & Y. Nakajima (Eds.), *Fechner Day 2017: The 33rd Annual Meeting of the International Society for Psychophysics* (p. 126). Fukuoka, Japan: International Society for Psychophysics.
